# Evaluating the Accuracy and Impact of the ESR-iGuide Decision Support Tool in Optimizing CT Imaging Referral Appropriateness

**DOI:** 10.1007/s10278-024-01197-5

**Published:** 2024-07-19

**Authors:** Osnat Luxenburg, Sharona Vaknin, Rachel Wilf-Miron, Mor Saban

**Affiliations:** 1 Medical Technology, Health Information and Research Directorate, Ministry of Health, Jerusalem, Israel; 2 The Gertner Institute for Health Policy and Epidemiology, Ramat-Gan, Israel; 3 Department of Health Promotion, School of Public Health, Faculty of Medical & Health Sciences, Tel Aviv University, Tel Aviv, Israel; 4 School of Health Professions, Faculty of Medical & Health Sciences, Tel-Aviv University, Tel-Aviv-Yafo, Israel

**Keywords:** Clinical decision support, CT scans, Decision tool, Radiology referrals, Referral quality

## Abstract

Radiology referral quality impacts patient care, yet factors influencing quality are poorly understood. This study assessed the quality of computed tomography (CT) referrals, identified associated characteristics, and evaluated the ESR-iGuide clinical decision support tool’s ability to optimize referrals. A retrospective review analyzed 300 consecutive CT referrals from an acute care hospital. Referral quality was evaluated on a 5-point scale by three expert reviewers (inter-rater reliability *κ* = 0.763–0.97). The ESR-iGuide tool provided appropriateness scores and estimated radiation exposure levels for the actual referred exams and recommended exams. Scores were compared between actual and recommended exams. Associations between ESR-iGuide scores and referral characteristics, including the specialty of the ordering physician (surgical vs. non-surgical), were explored. Of the referrals, 67.1% were rated as appropriate. The most common exams were head and abdomen/pelvis CTs. The ESR-iGuide deemed 70% of the actual referrals “usually appropriate” and found that the recommended exams had lower estimated radiation exposure compared to the actual exams. Logistic regression analysis showed that non-surgical physicians were more likely to order inappropriate exams compared to surgical physicians. Over one-third of the referrals showed suboptimal quality in the unstructured system. The ESR-iGuide clinical decision support tool identified opportunities to optimize appropriateness and reduce radiation exposure. Implementation of such a tool warrants consideration to improve communication and maximize patient care quality.

## Introduction

The quality of a referral or request for a radiologic examination is deemed as an important component to ensure the most appropriate imaging technique, to proficiently interpret the examination, to establish a differential diagnosis, and to provide appropriate information to the referring physician for further patient management. Incomplete or non-detailed referrals can have significant consequences for patients, radiologists, and the diagnostic process [1–3]. They can lead to delays in diagnosis and treatment, increase the risk of misdiagnosis, and result in inappropriate treatment. Radiologists heavily rely on the referral information provided by clinicians to interpret imaging studies accurately and formulate appropriate differential diagnoses. The quality and completeness of this referral information can have significant implications for the diagnostic process and patient management, as explored further in the following sections [3–5]. When referrals lack key details, it becomes challenging for radiologists to provide a proper diagnosis, leading to the need for additional imaging or consultations. This prolongs the diagnostic process, increases costs, and further risks misdiagnosis and inappropriate treatment, ultimately harming patient outcomes [5–7].

The referring physician plays a pivotal role in acting as a gatekeeper to supporting an efficient and a comprehensive diagnosis for the patient, and ultimately shortening clinical management pathways [8]. Given that radiologists have limited contact with a patient, the radiologist highly relies on the referrer’s own diagnostic process, which necessitates the synthesizing of medical history, examinations, and prior findings into a summarized formulation of the diagnostic question [4].

Previous literature has highlighted certain aspects of clinical information as being especially crucial and of value. Cohen [9] highlights the importance of identifying and orienting on a clinical question to be addressed, whereas Castillo et al. [10] has underscored that medical history of the patient improved the accuracy of CT reports, where in the context of more complex investigations, the more imperative the accuracy and completeness of clinical history of the patient. In fact, literature has highlighted that in the case of incomplete requisitions, where insufficient clinical information is provided to validate appropriateness, the result is uncertainty in warranting of imaging examinations and delays in diagnostic pathways [1, 11].

Previous investigations of the referral phenomenon for radiological investigations suggest insufficiencies with requests, where Depasquale et al. show that of 200 request forms reviewed, only 4% were completed in full and in 7% of cases clinical history was not presented [12]. Similarly, a study by Akinola of 145 imaging requests indicated that only 18% of cases provided a detailed clinical history [13].

To address these limitations, various guidelines and standardized systems have been introduced as interventions to improve the referral system to radiologists by stressing the need for adequate clinical details. For example, the ACR Appropriateness Criteria in the United States and the iRefer guidelines from the Royal College of Radiologists in the United Kingdom aim to improve the quality and appropriateness of imaging referrals [14–18]. However, the real-world effectiveness of these guidelines has been variable, with persistent issues of overutilization, underutilization, and inappropriate use of imaging studies [19–21].

In response to the limitations of the existing unstructured referral system, clinical decision support systems (CDSS) have been developed to optimize referral quality. For instance, the European Society of Radiology’s ESR-iGuide is a web-based decision support platform providing estimated costs and radiation exposure for the most suitable imaging exams. Studies have documented benefits such as improved guideline compliance and physician agreement through its use [22–24]. The literature demonstrates that CDSS can significantly increase adherence to imaging appropriateness recommendations. In response, several CDSS tools have been created, including the European Society of Radiology’s ESR-iGuide [25–27].

This underscores the need to explore innovative approaches, such as clinical decision support tools, that can optimize referral quality beyond the limitations of the existing unstructured referral system. In addition, direct interaction between clinicians and radiologists can facilitate better communication and collaboration among all the operators involved in patient management, and improve referral accuracy [28, 29].

A previous study aimed at assessing the quality and amount of involvement of radiologists in multidisciplinary tumor boards found that interaction with referring clinicians was perceived as having major benefits [29]. Despite improvements in radiology requisitions where decreased proportion of missing data is observed, findings still indicate that further improvements are necessary [16].

Accordingly, this study investigated the ESR-iGuide’s application and suitability within a single medical center, with the aim of understanding feasibility and potential benefits of broader national implementation across multiple centers.

### Study Aims

This study was designed to comprehensively evaluate the performance and utility of the ESR-iGuide decision support tool for optimizing radiology CT scan referrals. The ESR-iGuide is a well-validated reference standard based on the American College of Radiology’s Appropriateness Criteria, making it a robust tool for assessing the appropriateness of imaging orders [23, 30].

The primary objectives of this research were as follows:

1. To assess the quality and completeness of CT imaging referrals at a single healthcare center.
2. To identify factors that may influence variability in the appropriateness of CT referrals, such as referral source, clinical indication, patient demographics, and other relevant variables.
3. To evaluate the potential of the ESR-iGuide decision support tool to improve the appropriateness of CT imaging referrals.

### Hypotheses

The primary hypotheses guiding this study were as follows:

1. The appropriateness determinations made by the ESR-iGuide decision support tool will agree with those of independent radiologist expert reviews at a high level, demonstrating the tool’s accuracy and reliability in assessing the appropriateness of CT imaging referrals.
2. Specific factors, such as referral source, clinical indication, patient demographics, and other relevant variables, will be associated with variability in the appropriateness of CT referrals.

## Materials and Methods

### Data Collection

A retrospective study was conducted in 2022 in a secondary academic emergency Hospital setting in which approximately 6235 CT scans are performed annually. We estimate that approximately 575,000 in-hospital CT scans are conducted annually in Israel based on recently Ministry of Health data.

### Inclusion Criteria:

- All consecutive in-hospital CT scan cases ordered over a 4-week period at our secondary academic emergency hospital.
- Patients aged 18 years and older.

### Exclusion Criteria:

- Repeat/follow-up CT scans on the same patient during the study period (only the initial scan was included).
- Pediatric patients under the age of 18.

### Procedure

The study was conducted in two phases:

#### Phase 1—Assessing Referral Quality by Experts

For each case, we collected the original text referral, ordered test, patient characteristics (age, gender, clinical background), and physician characteristics (gender, specialty, status). Data was also gathered on the shift when the imaging test was performed and whether the image was interpreted as normal or abnormal.

Based on previous guidelines, [31, 32] the researchers developed a 5-point Likert scale to assess the quality and completeness of the referrals. The scale evaluated factors such as time dimension, description of symptoms, and inclusion of relevant clinical details. The time dimension referred to whether the referral specified the duration/onset of symptoms. A score of 1 indicated no relevant details were included, while a 3 meant the most important details (duration/onset of symptoms, relevant prior tests or treatments) were captured. A score of 5 represented a referral with a complete clinical history and timeline of present illness.

Scores of 2 and 4 represented intermediate levels of completeness. Two independent attending radiologists with over 10 years’ experience served as expert reviewers. Higher scores on the first three items (time dimension, symptoms, clinical details), with a maximum of 5 each, indicated higher quality referrals.

The latter two items assessed inclusion of non-critical information and unnecessary language, with lower scores representing higher quality (absence of superfluous content). Audio recordings were made of the reviewers scoring these items to ensure consistent interpretation and application of the scales. This process aimed to objectively evaluate referral quality for algorithm training and validation.

#### Phase 2-Assessing Referral Quality by ESR-iGuide

The patient cases were then entered into the ESR-iGuide clinical decision support platform. The appropriateness scores assigned by the tool to the ordered exam and recommended alternatives were recorded, using a 9-point scale. Appropriateness was rated on a 9-point scale, based on the criteria established by the ESR iGuide, where 7–9 is considered generally appropriate, 4–6 is possibly appropriate, and 1–3 is generally not recommended. The relative radiation levels of the exams were also obtained.

This two-phase process allowed for a comprehensive evaluation of the ESR-iGuide tool’s accuracy and ability to promote appropriate, evidence-based imaging referrals.

### Data Analysis

For the assessment of inter-rater reliability in phase 1, three physician experts from different medical specialties were enlisted to independently review and rate the 300 imaging referral cases. The three expert reviewers consisted of one board-certified emergency medicine specialist with over 15 years of experience evaluating clinical documentation, one public health and pediatrics specialist with a M.S. and 30 years of expertise in quality assurance processes, and one radiologist with 30 years of experience assessing patient referrals and medical records.

The three experts selected all have extensive experience in managing adult patient populations and regularly order CT scans as part of their clinical decision-making.

This multi-disciplinary panel of clinicians collectively reviewed the 300 cases using the 5-category grading scale developed for the study.

In the original scale, a higher score indicated poorer referral quality. However, to ensure a consistent interpretation where a higher score reflects better referral quality, we reversed the scales for items 4 and 5 prior to analysis. This harmonization process was documented in the methods section.

Referrals that received a score of 3 out of the total 5 points on our assessment tool were categorized as being of “good quality.” This indicated the referral contained the majority of the key information elements, such as the time dimension, symptom description, and relevant clinical details.

We then quantified the interrater reliability of individual item scores by using a Fliess Kappa coefficient. Based on the 95% confidence interval of the Fliess Kappa estimate, Fliess Kappa values of less than 0.5, 0.5 to 0.75, 0.75 to 0.9, and greater than 0.9 indicate poor, moderate, good, and excellent reliability, respectively [33]. We further calculated the mean and median ranking for each item across all 300 cases and built a variable that summed up all five mean variables.

Associations between item rating and each of the study variables were examined using *χ*^2^ tests for categorical variables and *t*-tests, Pearson correlation coefficient, or one-way ANOVAs, when appropriate, for continuous variables. The level of significance for all statistical analyses was 5%. The data analysis was performed using the Statistical Package for Health & Welfare Science for Windows (SPSS, version 28.0, Chicago, IL, USA).

### Sample Size

A sample size calculation was conducted. Based on a literature review, which evaluated previous accuracy of imaging referrals, we assumed a 20% inaccuracy rate [9, 27], with a confidence level of 95%. The sample size was calculated using OpenEpi software (Version 3.01), based on population size and statistical requirements for models of this type. Based on a frequency of 20%, test power of 80%, confidence interval of 95%, and significance of 0.05, there is a minimal sample size needed for 237 patients. A sample of 300 consecutive cases of CT imaging tests performed for in-patients from all hospital departments as well as the Emergency Department during 2021 were included in the final sample.

### Ethical Considerations

The study protocol was approved by the Institutional Human Subjects Ethics Committee (CM-0058-21) of the relevant medical facility. All procedures performed were following the ethical standards of the institutional and national research committee and also complied with national ethical standards.

## Results

Three hundred consecutive cases of imaging tests were included in the current study. The mean age of the patients was 59.96 ± 22.11, and majority of the patients were female (*n* = 175, 58.5%).

All cases were CT scans, usually as a single modality. Most of the CT exams were head CT (*n* = 200) as well as abdominal and pelvis CT (*n* = 76). Eighteen patients underwent more than one exam (CT and ultrasonography or CT and X-rays).

Most of the physicians were residents (*n* = 160, 53.5%) and 112 were senior physicians (*n* = 112, 37.5%). The leading specialties of the referring physicians were internal medicine, including sub-specialties such as neurology and nephrology (*n* = 95, 31.8%) and surgery/orthopedics (*n* = 97, 32.4%). Twenty-four referrals (8.0%) were made by emergency medicine physicians while 23 referrals (7.7%) were produced by interns. The most frequent shift when the exam was performed was the evening shift (*n* = 131, 43.8%) (Table 1).

**Table 1:** The study characteristics: patients, physicians, and shift characteristics

	*N* = 300
**Patient characteristics**	
Age (mean, SD)	59.96 ± 22.11
Gender—female (*n*, %)	175 (58.5)
**Clinical fields CT exam (*n*)**	
Head	200
Chest	33
Abdominal and pelvis	76
Musculoskeletal/Spine	3
Total body CT	5
**Medical team characteristics**	
Female (*n*, %)	111 (37.1)
**Physician status**	
Senior physician	112 (37.5)
Resident	160 (53.5)
Intern	24 (8)
**Physician specialty (*n*, %)**	
Internal medicine/neurology/nephrology	95 (31.8)
Surgery/orthopedics	97 (32.4)
Gynecology	2 (0.7)
Internship	23 (7.7)
Pediatrics	6 (2.0)
ENT	9 (3.0)
Otolaryngology	9 (3.0)
Emergency Medicine	24 (8.0)
**Shift characteristics** (*n*, %)	
Morning (7–15)	89 (29.8)
Evening (15–23)	131 (43.8)
Night (23–7)	49 (16.4)

The overall agreement between the three experts over the five items, calculated by Fleiss Kappa, ranged from good to excellent (0.763–0.97). These indices exceeded the recommended value of 0.70, providing justification for the aggregation [19].

**Fig. 1:**
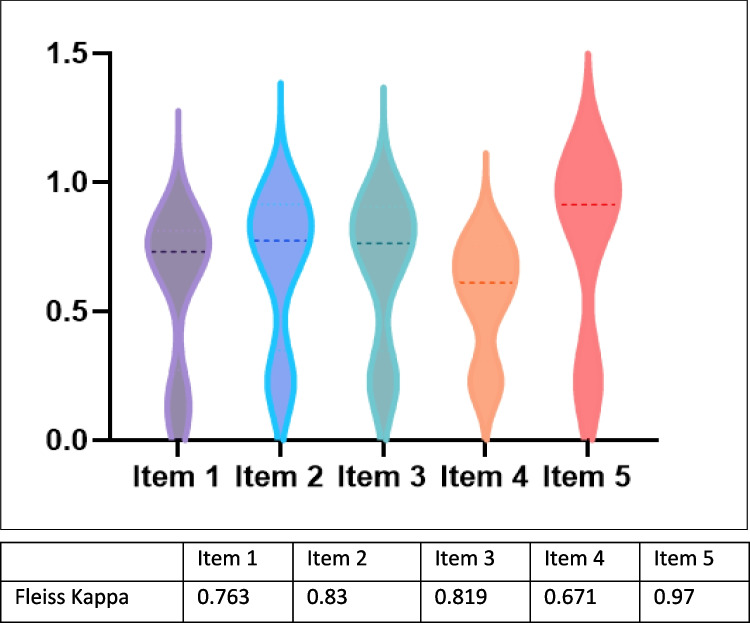
Inter-rated reliability, measured by Fleiss Kappa estimate

The mean and median ratings were high for four items (nos. 1, 2, 4, and 5) but relatively low for item no. 4 (this item indicates “unnecessary information”) (Fig. 1).

In 67.08% of the cases, the referral was of good quality. Item no. 2 (reference to the nature of the complaint) was rated as being of appropriate quality in 92% of the referrals examined while item no. 3 (treatment of background diseases/material clinical details) was indicated in the referrals as only in 34.1% of the referrals.

Referral quality did not demonstrate significant differences among the different medical specialties of the referring physician. No significant differences were found between referral quality. Referral quality during the morning, evening, and night shifts was statistically different on a five-level scale [3.48, 3.71, and 3.89, respectively (*p* = 0.025)].

Tables 2 and 3 show the descriptive statistics (mean, standard deviation) and the results of the gender comparisons (*p*-value, confidence intervals, effect size) for various aspects of the quality of radiology referrals, such as reference to the time dimension, the nature of the complaint, information on clinical background, provision of unnecessary information, and verbatim copying from medical records.

**Table 2:** The effect of patient gender on the quality of radiology referral

	**Patient gender**	**Mean**	SD*	***F***	***P* value**	Lower CI**	**Upper CI**	**Point estimate** **(Cohen’s *d*)**
Item1	Male	0.66	0.44	8.823	0.012	− 0.21	− 0.015	− 0.27
	Female	0.78	0.38					
Item2	Male	0.90	0.26	73.985	p < 0.001	− 0.13	− 0.037	− 0.47
	Female	0.98	0.08					
Item3	Male	0.44	0.47	0.778	0.076	− 0.19	0.03	− 0.17
	Female	0.52	0.48					
Item4	Male	0.86	0.29	11.264	0.011	0.010	0.151	0.27
	Female	0.78	0.32					
Item5	Male	0.73	0.44	18.876	0.021	0.004	0.216	0.24
	Female	0.62	0.49					
Overall items	Male	3.60	0.88	0.155	0.208	− 0.292	0.121	− 0.09
	Female	3.68	0.90					

Item 1 – reference to the time dimension; item 2 – reference to the nature of the complaint; item 3 – information on clinical background; item 4 – provision of unnecessary information; item 5 – information is copied “as is” from the medical records

*SD standard deviation

**CI confidence interval

**Table 3:** The effect of gender of the referring physician on the quality of radiology referral

	**Physician gender**	**Mean**	**SD**	***F***	***P* value**	**Lower CI**	**Upper CI**	**Point estimate** **(Cohen’s *d*)**
mean_item1	Male	0.67	0.44	39.22	*p* < 0.001	− 0.26	− 0.07	− 0.42
	Female	0.83	0.33					
mean_item2	Male	0.94	0.20	5.95	0.094	− 0.07	0.01	− 0.15
	Female	0.97	0.16					
mean_item3	Male	0.45	0.48	0.14	0.052	− 0.21	0.02	− 0.20
	Female	0.55	0.47					
mean_item4	Male	0.82	0.30	1.06	0.250	− 0.05	0.10	0.08
	Female	0.80	0.32					
mean_item5	Male	0.68	0.47	0.58	0.316	− 0.08	0.14	0.06
	Female	0.65	0.47					
Overall_items	Male	3.56	0.87	0.36	0.015	− 0.45	− 0.02	− 0.27
	Female	3.79	0.92					

Item 1 – reference to the time dimension; item 2 – reference to the nature of the complaint; item 3 – information on clinical background; item 4 – provision of unnecessary information; item 5 – information is copied “as is” from the medical records

*SD standard deviation

**CI confidence interval

The mean ratings for three of the five items (1, 2, 3) were significantly higher for female patients than males. Those items (reference to the time dimension, to the nature of the complaint and background clinical information) are the core of a good referral. This trend was reversed for items 4 and 5 (indication of unnecessary or redundant information; the information is copied “as is” from the medical records), with referrals of males’ patients ranked higher (Table 2).

The effect of gender on radiology referral quality was evident also for the gender of the referring physician. The mean rating for three items (1, 2, and 3), the female rating was higher as compared with male physicians.

The frequency of indicating unnecessary information and copying the information from the medical record was higher during the night shift compared to morning and evening [*F* (2, 266) = 6.644, *p* = 0.002; *F* (2, 266) = 5.558, *p* = 0.004, respectively].

The lowest rates of abnormal interpretation were found in imaging ordered by interns compared to specialists (*X*^2^ = 6.735, *p* = 0.036).

**Fig. 2:**
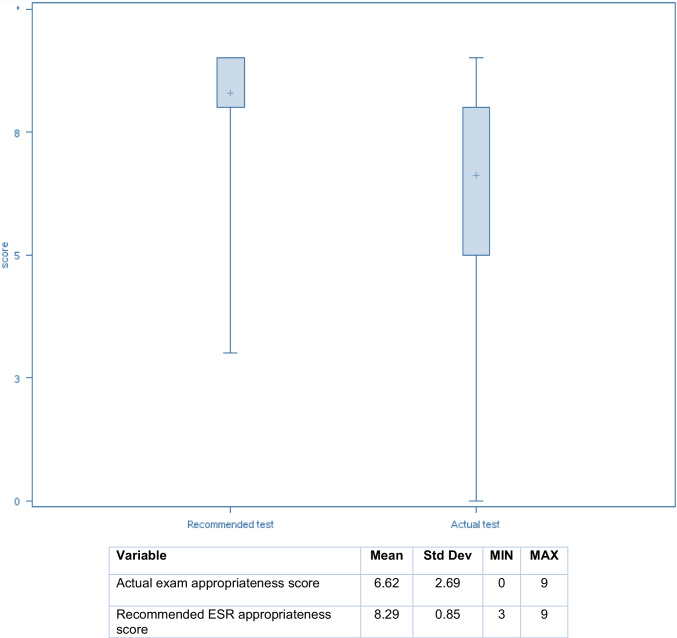
Box plots comparing ESR appropriateness scores between actual exams and ESR-iGuide’s best recommendations

The study also found that appropriateness scores assigned by the ESR-iGuide tool were significantly higher than scores for the originally referred exams. The mean appropriateness of actual referrals was 6.62 compared to 8.29 for the recommended alternative based on a 9-point scale (Fig. 2).

**Fig. 3:**
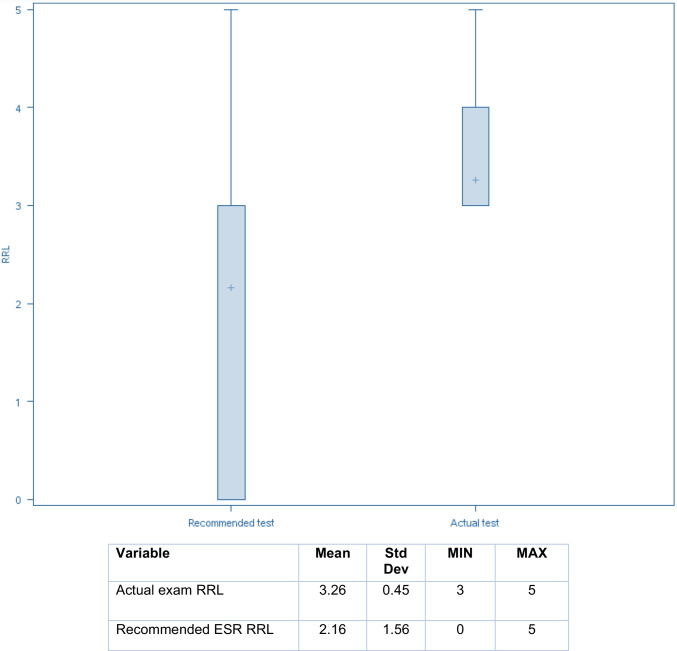
Box plots comparing ESR radiation levels between actual exams and ESR-iGuide’s best recommendations

Relative radiation levels were also significantly lower for ESR-iGuide recommendations, with a mean of 2.16 versus 3.26 for referrals (Fig. 3).

When appropriateness was categorized as generally appropriate (scores 7–9) or may/not appropriate, 70% of referrals were in the former group. Additionally, a significant association was found between physician specialty (surgery vs. non-surgery) and ESR-iGuide appropriateness scores. Analysis also revealed surgery referrals were less likely to match ESR-iGuide recommendations.

## Discussion

The results of this study provide important insights into optimizing the quality of radiology referrals through the use of clinical decision support.

As indicated in previous studies, around 20–30% of referrals were deemed inappropriate or of suboptimal quality based on the ESR-iGuide assessment. This is consistent with previous research that has documented inadequate referral quality in other contexts [14–17]. A notable finding revealed that in 34% of cases, clinical details were simply copied from records without a clear diagnostic question. Inadequate referral quality can negatively impact patient management through misdiagnosis or delays [22, 34, 35].

This study also found approximately 30% of CT exams were defined as “may be appropriate/usually not appropriate” according to the ESR-iGuide, mainly involving head and abdominal CTs. This is similar to rates reported in other studies assessing unnecessary imaging [36, 37].

A recent study from 2022, using the ESR-iGuide, assessed the appropriateness of CTs for acute abdominal pain. The findings showed that according to the ESR-iGuide and based on the clinical suspicion of CT requests, CT examination were considered crucial in 264 (45.05%). 54.9% of the patients had a referral reason for CT exam that could be considered “may be appropriate” according to ESR-iGuide criteria (4, 5, 6 scoring). Of these, 135 had an inappropriate CT request according to image findings [38]. As defined by Ruhm et al., the inappropriate imaging rate was around 25% when accounting for margin of error, leading to inadequate resource use and patient radiation exposure [39]. The current study also found significantly higher radiation exposure for actual exams compared to ESR-iGuide recommendations. Physicians have generally shown interest in controlling patient radiation exposure from overuse of CT exams [39, 40].

However, we did not find any other significant associations between imaging exam appropriateness and other patient-level factors such as age or time of day ordered. This suggests that while there may be some systematic gender-based differences in certain aspects of referral quality, the overall appropriateness of imaging requests does not appear to be driven by broader biases related to patient demographics or provider cognitive load during off-hours.

These findings indicate that the differences in referral quality we identified are more likely due to factors specific to the clinical decision-making process, rather than easily measurable patient or provider characteristics. Further research would be needed to explore the potential underlying causes of the observed gender disparities in certain referral quality metrics.

These null findings are noteworthy, as they challenge some common assumptions about potential drivers of variability in referral quality. The absence of associations with patient age, gender, or shift timing implies that the observed differences in imaging appropriateness are more likely attributed to provider-level factors, such as specialty-specific training and familiarity with clinical guidelines, as discussed earlier.

Exams ordered by training physicians had a non-significantly higher appropriate rate than specialist exams. Evolving evidence suggests specialists and novices employ different decision-making approaches, with specialists relying more on intuition while novices follow analytic protocols [41].

Our study focused on the quality of imaging referrals and their impact on the appropriateness of ordered CT scans. While we did not directly examine the use of clinical decision support systems, the literature suggests these tools can play an important role in optimizing imaging utilization [22, 30, 34, 42].

Decision support systems that provide guidance on appropriate imaging orders have been shown to help reduce unnecessary testing and improve alignment with evidence-based guidelines. However, these decision support tools are most effective when used in conjunction with the clinical expertise and intuitive judgment of the ordering provider [25, 43]. Physicians often rely on a combination of objective data and subjective clinical judgment when deciding on the most appropriate course of action for a patient. Decision support can supplement this process by flagging key clinical factors and providing recommendations. But the final decision should balance the output of the decision tool with the provider’s own assessment of the unique needs and circumstances of the individual patient. Surgeons ordered appropriate exams at a higher rate than other specialists, while internal medicine providers faced less defined clinical presentations, possibly due to dealing with more concrete protocols for conditions like appendicitis. The higher rate of appropriate imaging orders among surgeons compared to other specialty providers may be attributed to the more standardized, protocol-driven nature of surgical care for certain acute conditions. For example, the workup and management of appendicitis follows well-established clinical guidelines that clearly outline the appropriate use of imaging modalities like CT scans. In line with our results, Young et al. found that primary care physicians (internal medicine, family medicine) were almost twice as likely to order an inappropriate MRI as orthopedists, neurologists, and surgeons [44].

Future studies could focus on cases of disagreement between physicians and the ESR-iGuide to better inform system improvements and physician guidance. Evaluation of similar decision support tools has found most canceled referrals were appropriately flagged, though a minority of cases proved clinically significant and still required imaging. Both physician and system limitations need consideration to optimally manage resources and patient care [45].

By evaluating the referrals against the ESR-iGuide decision support tool, which is based on the established American College of Radiology Appropriateness Criteria, we were able to assess the quality and appropriateness of the imaging referrals made by providers. While a meaningful portion of referrals were deemed appropriate on initial review, the decision support tool identified opportunities to enhance appropriateness and lower unnecessary radiation exposure in the majority of cases through alternative recommended exams. Areas of discrepancy highlighted how decision support could help guide referrers, whereas concordant appropriate referrals demonstrated current best practices. Factors associated with lower quality referrals provide targets for targeted interventions. Overall, the results support integrating clinical decision support software like the ESR-iGuide to systematically and objectively evaluate referrals, help standardize communication between referrers and radiologists, and promote high-value, optimized radiological imaging through identification of unnecessary or suboptimal examinations.

### Limitations

Our study has limitations. First, the study focused on patients undergoing head and abdominal CTs at a single institution, limiting generalizability to other settings or patient populations with different imaging rates. Results may not apply to other imaging tests or healthcare systems. Second, the retrospective design may introduce bias and limit establishing causality. Retrospective studies rely on existing data, which may lack relevant variables or account for confounding factors. Despite these limitations, the study offers valuable insights into referral quality for imaging tests and highlights areas for improvement.

## Conclusions

Evaluating the quality of radiology referrals is imperative to ensure optimal patient care and resource utilization. As demonstrated in this study, a significant portion of referrals exhibited suboptimal documentation of clinical details important for accurate radiological interpretation. Inadequate referrals can negatively impact diagnosis and management. However, referral quality is complex to assess and various factors likely influence documentation practices. This study contributes novel insights by assessing quality via both the referrals themselves, as reviewed by experts, as well as through the ESR-iGuide clinical decision support system. The ESR-iGuide offered an objective screening of appropriateness based on standardized criteria. Its evaluation revealed opportunities to improve appropriateness and reduce unnecessary radiation exposure from referrals. Comparing the two quality assessment methods provides a more comprehensive picture of strengths and limitations in the current referral process. Areas of agreement and discordance between the approaches also offer insight into optimizing referral guidance and decision support tools going forward.

## Data Availability

The analyzed data will be made available to requesting researchers upon a reasonable request.
